# Establishment of porcine enterocyte/myofibroblast co-cultures for the growth of porcine rota- and coronaviruses

**DOI:** 10.1038/s41598-018-33305-1

**Published:** 2018-10-12

**Authors:** Tingting Cui, Sebastiaan Theuns, Lowiese M. B. Desmarets, Jiexiong Xie, Gaëtan M. A. De Gryse, Bo Yang, Wim Van den Broeck, Hans J. Nauwynck

**Affiliations:** 1 Department of Virology, Parasitology and Immunology, Laboratory of Virology, Faculty of Veterinary Medicine, Ghent University, Salisburylaan 133, B-9820 Merelbeke, Belgium; 2 Department of Morphology, Faculty of Veterinary Medicine, Ghent University, Salisburylaan 133, B-9820 Merelbeke, Belgium

## Abstract

A stable culture of primary porcine enterocytes is necessary to study porcine enteric virus replication characteristics. Because the direct cultivation of primary porcine enterocytes is difficult, alternatives have to be considered. As subepithelial myofibroblasts secrete extracellular matrix and growth factors contributing to the attachment, proliferation and differentiation of epithelial cells, co-cultures of primary porcine enterocytes (ileocytes and colonocytes) with myofibroblasts were developed and evaluated for their susceptibility to enteric viruses. First, it was demonstrated that the co-cultured ileocytes and colonocytes were susceptible to an archival rotavirus strain RVA/pig-tc/BEL/RV277/1977/G1P[7] and different other rotavirus genotypes (fecal samples containing G5P[7], G5P[13], G9P[23], G4P[6]). Next, the TGEV Purdue strain infected both ileocytes and colonocytes whereas the Miller strain only infected ileocytes. Last, the PEDV CV777 Vero adapted and non-adapted (fecal suspension) strains could infect co-cultured ileocytes but not colonocytes. The infectivity of the CV777 Vero adapted strain was higher when the cells were cultured without fetal bovine serum and the CV777 fecal suspension only infected the ileocytes cultured without fetal bovine serum. In conclusion, a novel co-culture of porcine enterocytes with myofibroblasts was established, which can be used for the investigation of the replication of enteric viruses.

## Introduction

Enteric viruses are common causes of diarrhea in humans and animals. Porcine rotavirus, transmissible gastroenteritis virus (TGEV) and porcine epidemic diarrhea virus (PEDV) are well known enteric viruses, leading to high morbidity and mortality in piglets and causing economic losses in swine-producing countries. Rotavirus belongs to the genus rotavirus within the family *Reoviridae*. It consists of a triple-layered capsid encapsulating a genome consisting of eleven segments of double-stranded RNA (dsRNA) that encodes six structural (VP1-VP4, VP6 and VP7) and six non-structural (NSP1-NSP6) proteins. According to the inner capsid protein VP6, 10 different species/groups (A-J) of rotavirus have been identified using a 53% amino acid cut-off value sequence classification system^1–3^. Rotavirus group A, B, C, E and H have been detected in pig feces^4–8^. The glycoprotein VP7 and the protease-sensitive protein VP4, which elicit neutralizing antibodies, form a genotyping system within the rotavirus A species^9^. Until now, 35 G-genotypes (VP7) and 50 P-genotypes (VP4) were identified. The most prevalent rotavirus group A (RVA) strains in pigs are G3, G4, G5, G9, and G11 in association with P[6], P[7] P[13] and P[23] and the most predominant genotype combination among porcine RVA strains is G5P[7] worldwide^10,11^. Transmissible gastroenteritis virus and porcine epidemic diarrhea virus belong to the group I coronaviruses. They are enveloped viruses with an approximately 28.5 kb single-stranded, positive-sense RNA genome encoding four structural proteins: the spike (S), membrane (M), envelope (E), and nucleoprotein (N) protein. The spike protein is the major target for neutralizing antibodies. It mediates the virus binding to the cell surface receptor aminopeptidase N and plays a role in the fusion between the viral envelope and the cell membrane^12,13^. The nucleoprotein wraps the virus genome and supports virus assembly^14^. The membrane protein is mostly embedded in the lipid membrane and plays an important role in the virion architecture^15^. They cause lethal watery diarrhea and dehydration in piglets and destroy villous enterocytes in the small intestine. The severity and lethal outcome are strain dependent^16^. Different non-intestinal cell lines have been used in the past for virus cultivation *in vitro*^17,18^, but at low efficiency. TGEV can be propagated in swine testicle (ST) and porcine kidney (PK-15) cells. Upon adaptation, PEDV may infect African green monkey kidney (Vero) cells. Like TGEV and PEDV, rotavirus is mainly transmitted by fecal-oral route and virus infection causes the destruction of mature small intestinal enterocytes. Similarly, most rotavirus research was conducted on non-polarized MA104 cells (African green monkey kidney epithelial cells) which are easy to culture and permissive for certain rotavirus strains of different genotypes. But there are still a lot of genotypes of rotavirus strains that do not grow in MA104 cells^8^. Data concerning the replication cycles of these enteric viruses in their target cell (mature intestinal enterocytes) are scarce. To this end, it is essential to obtain cultures of porcine intestinal enterocytes.

The gastrointestinal tract is lined with a rapidly proliferating simple columnar epithelium. The epithelial cells migrate from the crypts where mitosis takes place towards the top of the villi (small intestine) or towards the top of the intercrypt (large intestine) as they mature. Mature epithelial cells are replaced by a steady supply of crypt cells. In suckling piglets, intestinal epithelial cells renew every 2–3 days. Because of the highly dynamic and rapid renewal properties of intestinal epithelial cells, it is difficult to successfully culture them *in vitro*. In 2014, a porcine mid-jejunum epithelial cell line was established from neonatal piglets by immortalization upon transfection/transduction with human telomerase reverse transcriptase (hTERT) gene^19^. Because the immortalization alters the biology of the original cells, field viruses replicate more efficiently in primary cells than in continuous cell lines. Therefore, it is advisable to use primary cells for virus research. Intestinal enteroids have been developed by culturing intestinal crypts onto Matrigel which is enriched with laminin α1 and α2^20^. Enteroids enhanced the viability of the cells and were already used for the study of rotaviruses^21^, noroviruses^22^ and enteroviruses^23^. The successful cultivation of enteroids is dependent on many growth factors, critically including Wnt 3a, R-spondin, and Noggin, which is an expensive method.

Intestinal myofibroblasts, one of the intestinal mesenchymal cells, are directly subjacent to the basement membrane of epithelial cells and they have been reported to support the proliferation and differentiation of epithelial cells^24^. Myofibroblasts are identified by the expression of intracellular cytoskeletal microfilament α-smooth muscle actin (α-SMA). Myofibroblasts contribute to the growth and differentiation of intestinal enterocytes by secreting several growth factors (hepatocyte growth factor, transforming growth factor beta (TGF-β1)^25,26^, insulin-like growth factors (ICFs)^24^), extracellular matrix proteins (collagen type IV, laminin-β1 and γ1, and fibronectin), cytokines and chemokines. To date, a mouse colonic myofibroblast cell line established by Hirokawa has been reported to stimulate colonoid formation^27^ and human myofibroblasts isolated from small intestine were able to support human intestinal epithelial cell growth *in vitro*^28^. All the information suggested the potential role of myofibroblasts in intestinal enterocytes cultivation *in vitro*.

In this study, a porcine co-culture system of primary intestinal enterocytes with intestinal myofibroblasts was established which mimics the enterocytes growth *in vivo*. The morphological and functional features of co-cultured enterocytes were characterized. To determine the usability of this co-culture system, enteric rota- and coronaviruses were used to infect the co-cultured enterocytes.

## Results

### Localization of epithelial cells and myofibroblasts in ileum and colon of a three days old piglet

**Figure 1:**
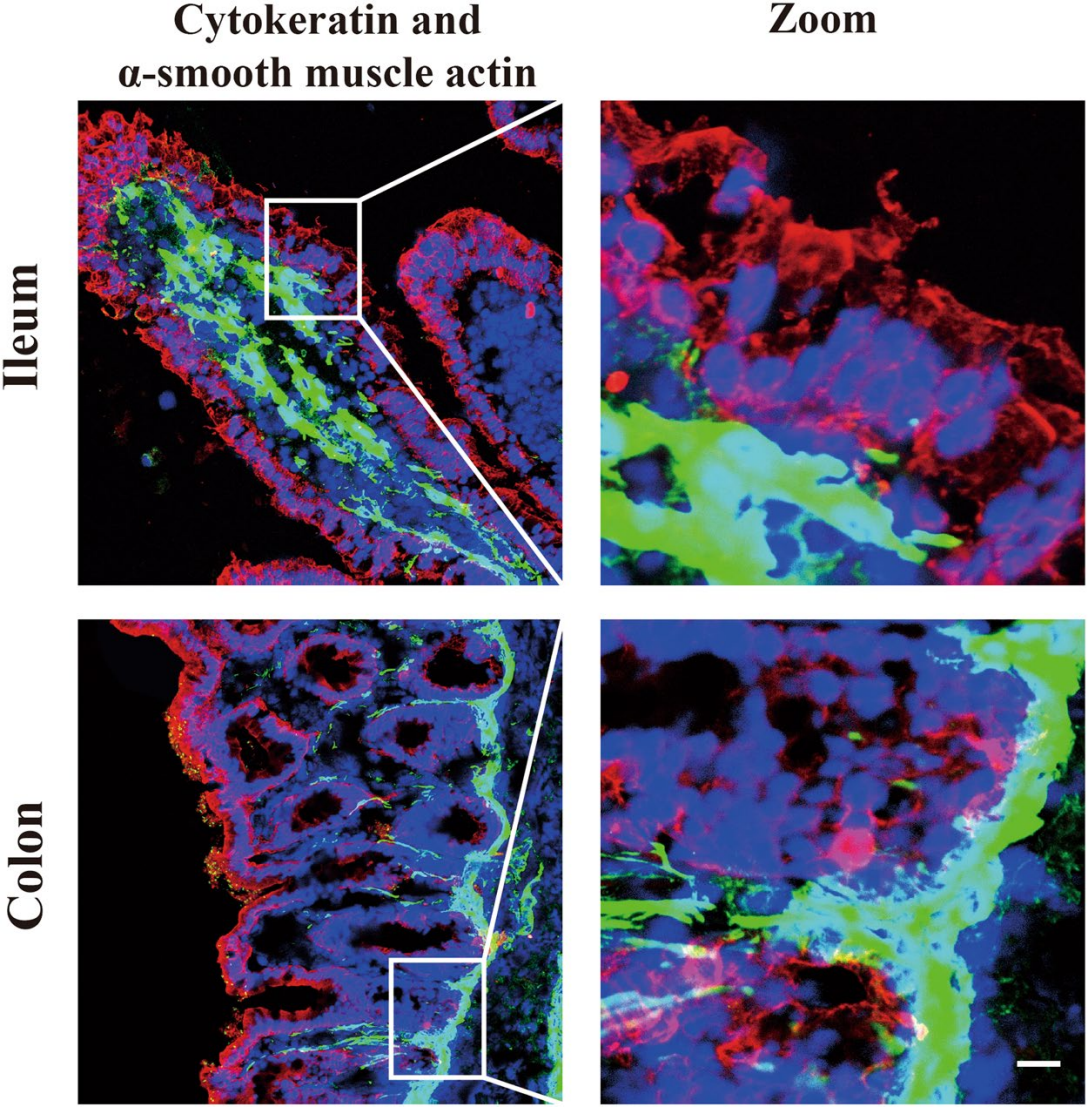
Characterization of the epithelial cells and myofibroblasts of the ileum and colon of a 3 days old piglet. Immunofluorescence staining against cytokeratin and α-smooth muscle actin in ileum and colon cryosections. A lot of myofibroblasts are located in the lamina propria underneath the epithelial layer of both tissues. Scale bar: 10 µm.

After euthanasia of a three-day old piglet, ileum and colon tissues were embedded immediately and cryosections were made to visualize the distribution of epithelial cells and myofibroblasts *in vivo*. Immunofluorescence stainings were performed against the epithelial cell marker cytokeratin and myofibroblast marker α-smooth muscle actin. In the porcine ileum, a lot of myofibroblasts were located in the lamina propria directly underneath the epithelial cell layer of the villi, representing the largest cell population in the lamina propria. Fewer myofibroblasts (<5%) were observed in the lamina propria underneath the epithelial cell layer in colon. In contrast, the myofibroblasts formed an integral line underneath the epithelial cell layer in the colon crypts (Fig. 1). These results show that porcine small and large intestinal epithelial cells grow in close contact to myofibroblasts *in vivo*. The contact communication between these two cell types is supposed to be an important element for the support of myofibroblasts in the attachment, proliferation and migration of epithelial cells.

### Isolation and characterization of ileum subepithelial myofibroblasts

**Figure 2:**
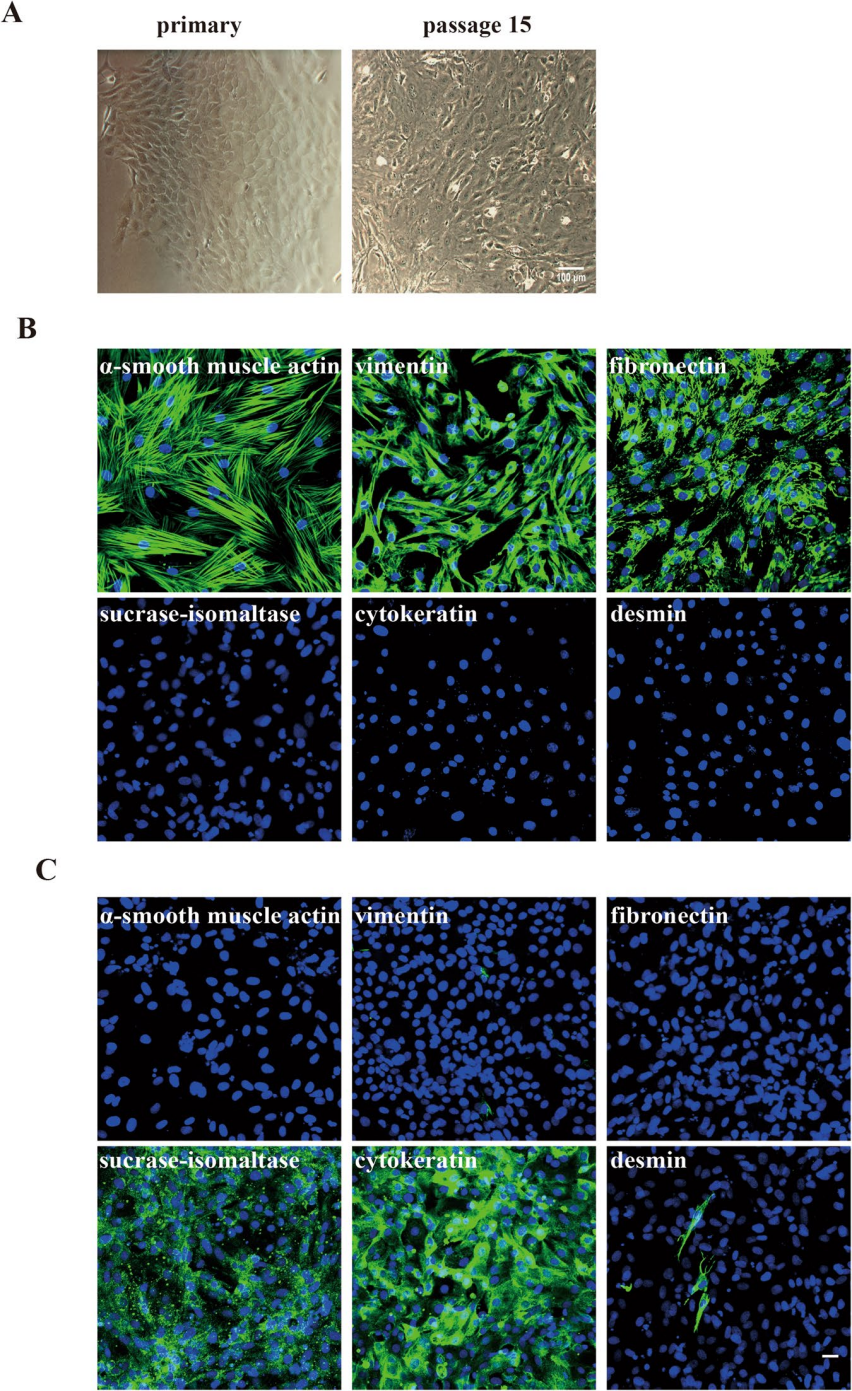
Morphological characterization of the myofibroblasts isolated from porcine ileum. (**A**) Characteristics of porcine ileum myofibroblasts. Myofibroblasts could be observed at 5 days after isolation. They had a cobblestone like morphology. They could be further sub- passaged as a continuous cell line. (**B**) A third passage of myofibroblasts was identified by immunofluorescence staining. Cells stained characteristically for intestinal myofibroblasts. They were positive for α-smooth muscle actin, vimentin and fibronectin, while cells were negative for sucrase-isomaltase, cytokeratin and desmin. (**C**) Primary ileum epithelial cells were used as control and stained with all markers that were used for the staining of the myofibroblasts. Scale bar: 25 µm.

Myofibroblasts were isolated from the subepithelial layer of porcine ileum. Cell clusters were observed 5 days post isolation and had a cobblestone-like morphology with stellate edges. The clusters continued to grow into larger structures which contain more than 200 cells. Meanwhile, the fibroblasts started to expand. The biggest cluster was marked by making a circle with a pen on the bottom of the plate and other cells were scraped away using a sterile tip. Afterwards, this circled cluster was split using trypsin. Cells maintained their morphology and could be continuously passaged (Fig. 2A). The obtained cells were characterized by immunofluorescence staining. Cells were stained positive for α-smooth muscle actin (α-SMA), vimentin and fibronectin, which are all markers of myofibroblasts. The cells were negative for desmin, cytokeratin and sucrase-isomaltase (Fig. 2B). These results confirmed that the cells isolated from the ileum subepithelial layer were myofibroblasts. Primary ileum epithelial cells were used as control, which were positive for cytokeratin and sucrase-isomaltase (Fig. 2C).

### Myofibroblasts serve as supporting cells for porcine enterocytes

**Figure 3:**
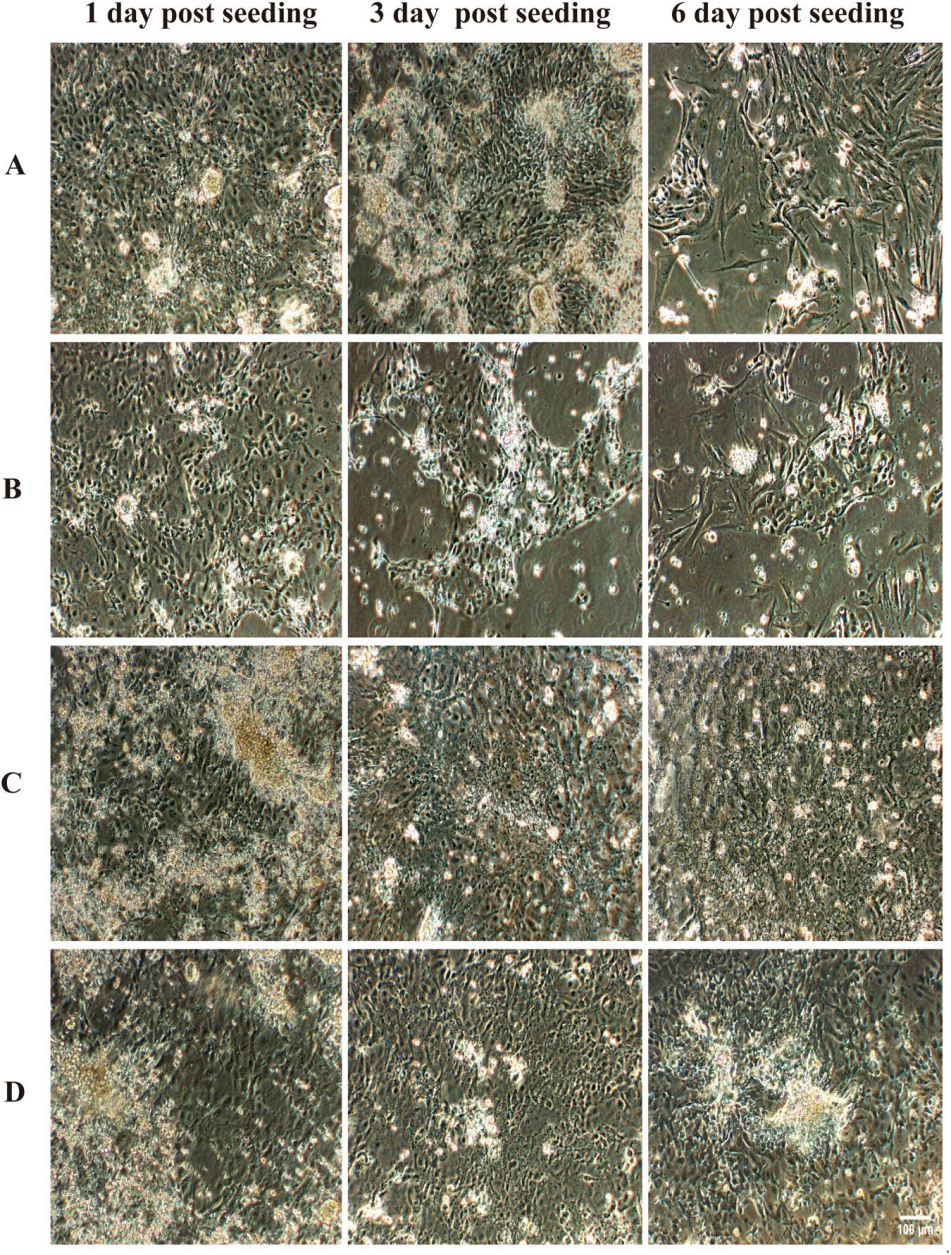
Evaluation of different methods to support the growth of primary porcine ileum and colon epithelial cells. (**A**) Porcine ileum epithelial cells were cultured without myofibroblasts on a porcine collagen type I/III coated plate. They could live approximately 2 days before detaching and dying, fibroblasts took over 6 days after seeding. (**B**) Porcine ileum epithelial cells were cultured without myofibroblasts on a porcine collagen type I/III coated plate with medium supplemented with 20% conditioned medium collected from ileum myofibroblasts. Epithelial cells could be maintained for less than 3 days and a few tiny epithelial clusters were present at 6 days post seeding. Porcine ileum (**C**) and colon (**D**) epithelial cells were cultured in the presence of ileum myofibroblasts. Epithelial cells grew into a monolayer 3 days post co-cultivation and maintained their morphology for more than 6 days.

Twenty-four hours post seeding, most of the ileum epithelial cells were attached and became confluent (>90%) when seeded on porcine type I and III collagen coated wells. However, 2 days post seeding, the epithelial cells started to detach, and 3 days post seeding, most of the epithelial cells were dead. Only a few big epithelial cell clusters could be visualized. Six days post seeding, fibroblasts took over the wells and a few tiny epithelial clusters were still present (Fig. 3A). The use of 20% conditioned medium did not give an improvement: most of the epithelial cells were dead at day 3 (Fig. 3B). When monolayers of myofibroblasts were used as supporting layers for ileum and colon epithelial cell cultivation, epithelial clusters attached to the myofibroblasts and became visible at 24 hours post seeding. The epithelial cells continued to expand and grew into monolayers 3 days post isolation. They maintained their polygonal morphology and confluent layers for more than 1 week (Fig. 3C,D).

### Characterization of enterocytes co-cultured with myofibroblasts

**Figure 4:**
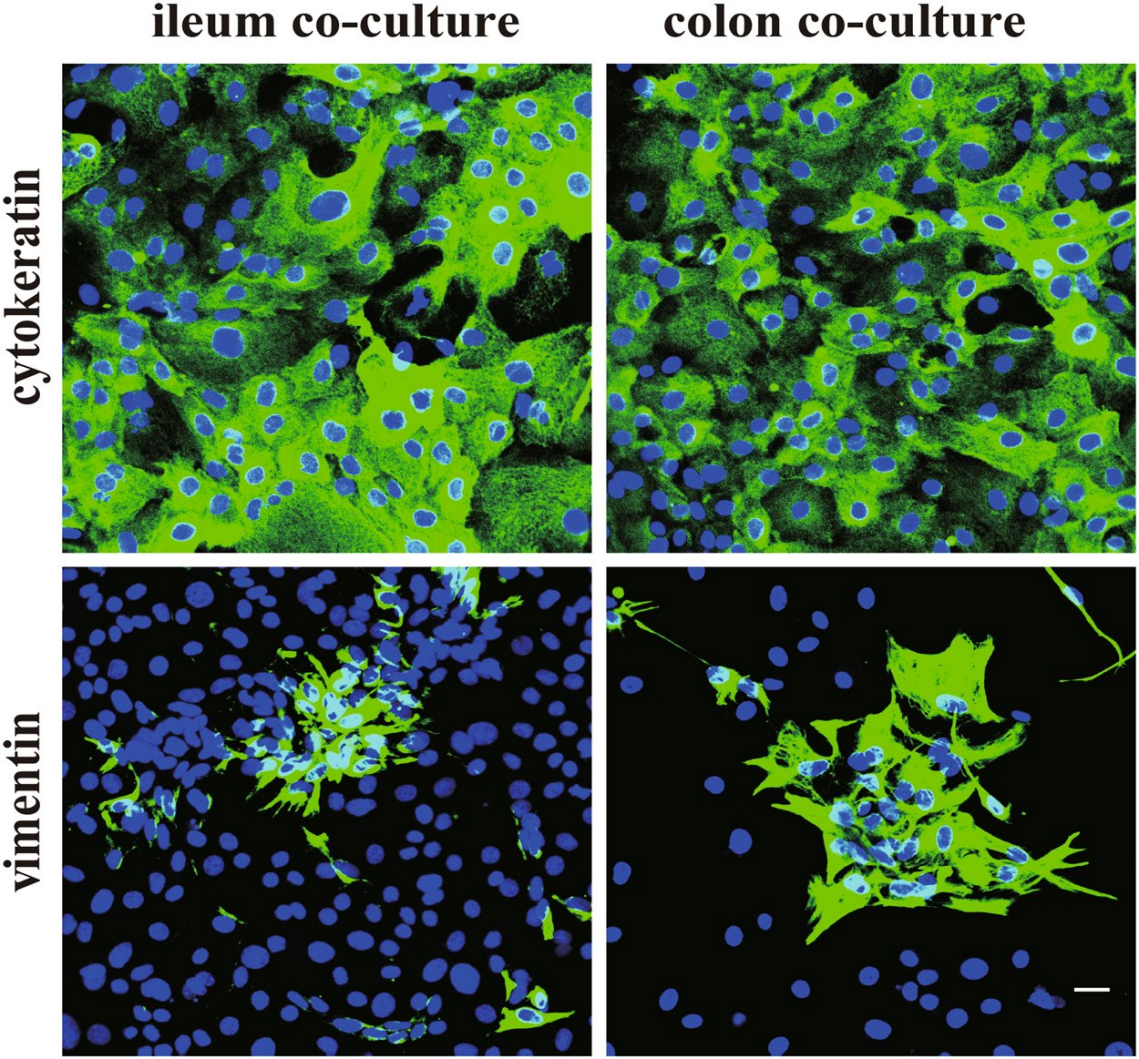
Characterization of the primary porcine ileum and colon epithelial cells co-cultured with ileum myofibroblasts. Immunofluorescence staining against cytokeratin and vimentin for ileum and colon epithelial cells co-cultured with myofibroblasts 3 days post seeding. Scale bar: 25 µm.

Cells isolated from ileum and colon co-cultured with myofibroblasts were characterized by immunofluorescence staining 3 days post co-cultivation. Antibodies against cytokeratin and vimentin were used to determine the epithelial nature. In the co-culture system, most of the cells were found to be cytokeratin positive (95 ± 1.9% of all cells in the ileum epithelial cell co-cultures and 91 ± 3.6% of all cells in the colon epithelial cell co-cultures), confirming the epithelial nature of the polygonal cells (Fig. 4). Interestingly, the vimentin positive cells (myofibroblasts) clustered into aggregates in both co-cultures, which suggests that the expansion of epithelial cell growth squeezed the myofibroblasts into aggregates.

### Scanning electron microscopy of epithelial cells of small and large intestines (*in vivo*) and after co-culture with myofibroblasts (*in vitro*)

**Figure 5:**
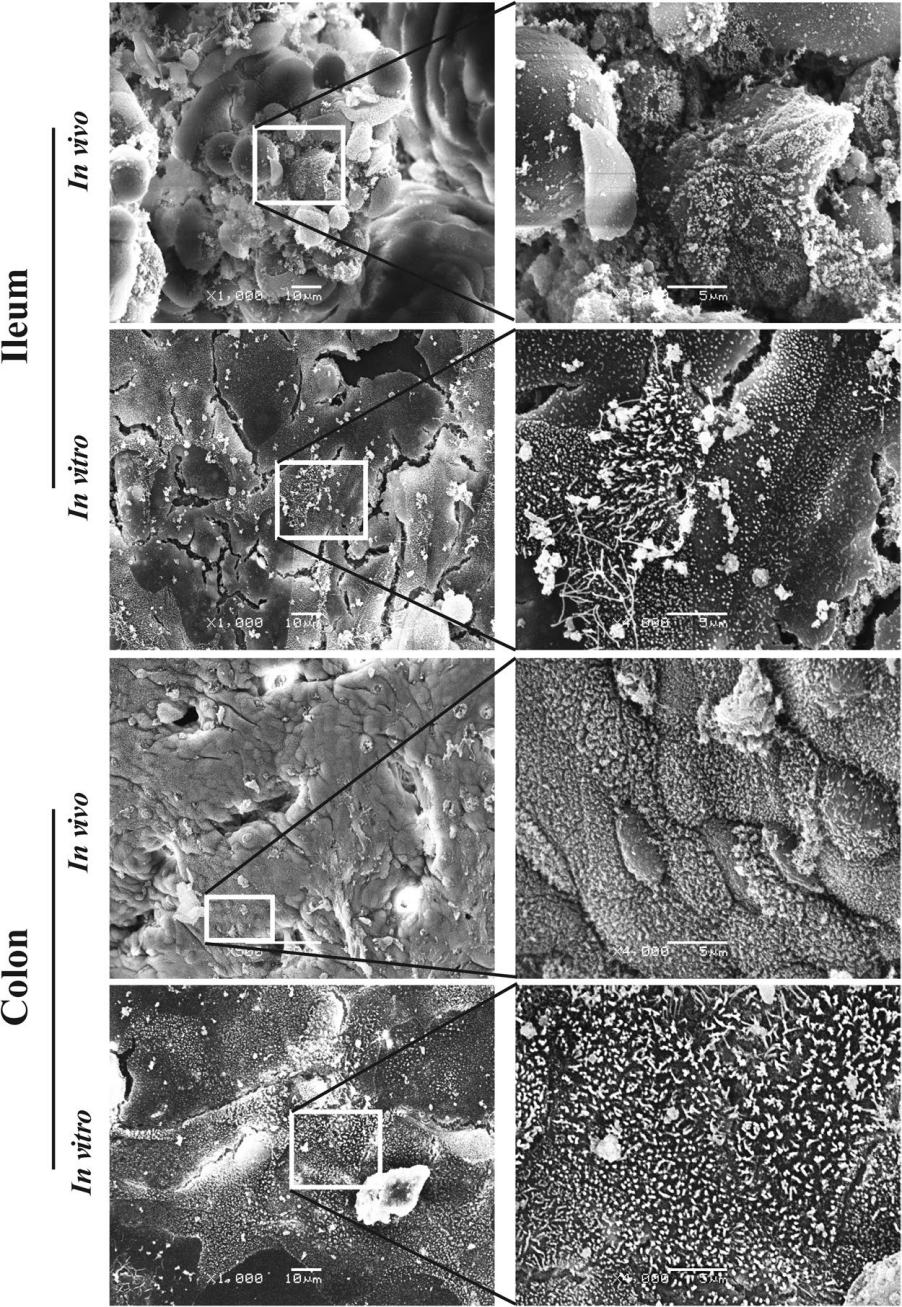
Scanning electron micrographs of ileum and colon epithelial cells *in vivo* and *in vitro*. Microvilli are present on a few epithelial cells at the top of an ileum villus of a 3 days old piglet. Cultured ileum epithelial cells are covered with microvilli. In the colon of a 3 days old piglet, almost all the epithelial cells were covered with short microvilli. A large number of microvilli were present on the colon epithelial cells at 3 days post seeding.

The differentiation status of porcine intestinal epithelial cells of 3 days old piglets and the co-cultured enterocytes were analyzed by scanning electron microscopy. As shown in Fig. 5, the epithelial cells of the ileum and colon had a different appearance *in vivo*. A few epithelial cells on the tip of a villus of the ileum were fully covered with microvilli, while most of the cells were immature without microvilli on the surface. In contrast, almost all epithelial cells of the colon had short microvilli. In the co-cultured ileum and colon epithelial cells, a lot of microvilli were present on the cell’s surface at three days post co-cultivation and showed a different appearance (some microvilli were longer). These results demonstrate that myofibroblasts could support the differentiation of both ileum and colon epithelial cells *in vitro*.

### Replication kinetics of rotavirus RVA/Pig-tc/BEL/RV277/1977/G1P[7] strain in co-cultured enterocytes

**Figure 6:**
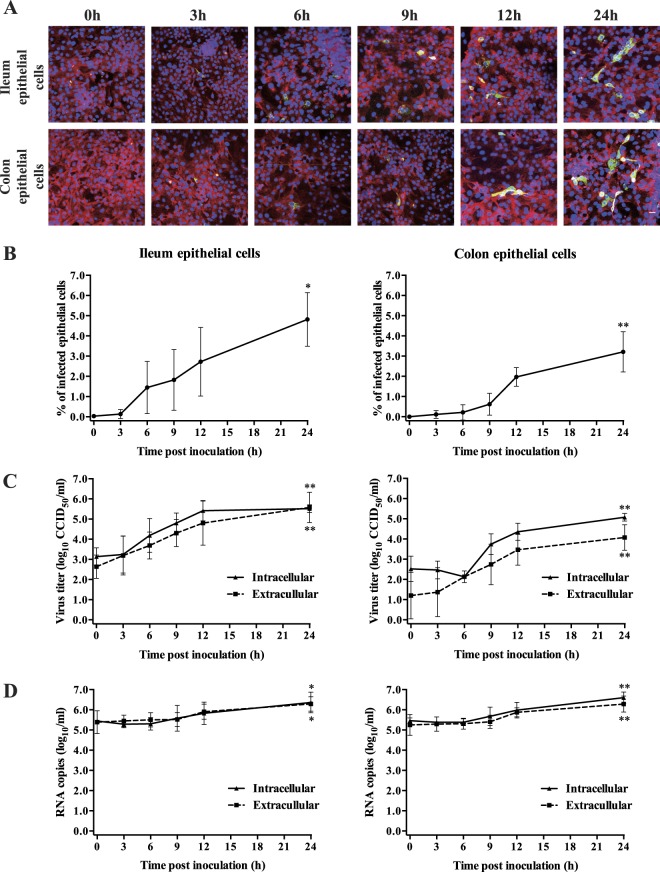
Kinetics of rotavirus replication in primary ileum and colon epithelial cells co-cultured with myofibroblasts. Three days post co-cultivation, cells were inoculated with rotavirus RVA/pig-tc/BEL/RV277/1977/G1P[7] strain at an m.o.i. = 1. At different time points post inoculation, (**A**) infected cells were visualized by immunofluorescence staining (red represents cytokeratin and green represents rotaviral antigen positive cells), (**B**) the percentage of infected epithelial cells was determined, (**C**) intra- and extracellular virus titers were assessed and (**D**) intra- and extracellular viral RNA loads were quantified by RT-qPCR. Scale bar: 25 µm. Data are expressed as mean ± standard deviation of the results of 3 separate experiments. Statistically significant differences in comparison with the data from 0 h p.i. are represented as *P <0.05 or **P <0.01.

To determine the percentage of rotavirus infected cells in primary porcine enterocytes, cells were fixed at different time points (0, 3, 6, 9, 12, 24 h) post inoculation with a low-passage archival RVA strain. Viral antigens were stained by immunofluorescence (Fig. 6A). The first antigen-positive ileum epithelial cells appeared at 6 h p.i and increased over time. The percentage of rotavirus infection in ileum epithelial cells increased to 4.8 ± 1.3% at 24 h p.i. In colon epithelial cells, the first antigen-positive cells appeared at 9 h p.i and the percentage of infection increased to 3.2 ± 0.9% at 24 h p.i (Fig. 6B). To determine the kinetics of virus production in ileum and colon epithelial cells, viral titers and RNA copies of supernatant (extracellular virus) and cells (intracellular virus) were assessed (Fig. 6C,D). The intracellular and extracellular virus titers were determined from 0 to 24 h post inoculation. The intracellular virus titer of ileum epithelial cells increased from 10^3.1±0.4^ CCID_50_/ml to 10^5.5±0.2^ CCID_50_/ml. The extracellular virus titer of ileum epithelial cells increased from 10^2.6±0.7^ CCID_50_/ml to 10^5.6±0.5^ CCID_50_/ml. For colon epithelial cells, the intracellular and extracellular virus titers increased from 10^2.5±0.6^ CCID_50_/ml to 10^5.1±0.2^ CCID_50_/ml and from 10^1.2±1.1^ CCID_50_/ml to 10^4.1±0.6^ CCID_50_/ml, respectively. The RT-qPCR showed that viral RNA started to be synthesized in ileum and colon epithelial cells from 9 h p.i. Viral RNA increased up to 6.4 ± 0.5 log_10_/ml in ileum epithelial cells and 6.6 ± 0.3 log_10_/ml in colon epithelial cells. The viral RNA started to be released into the supernatant between 9 and 12 h p.i; at 24 h p.i, 6.3 ± 0.4 log_10_/ml RNA copies were detected in both ileum and colon epithelial cell cultures.

### Susceptibility of primary porcine enterocytes to different rotavirus genotypes present in fecal suspensions of diarrheic pigs

**Figure 7:**
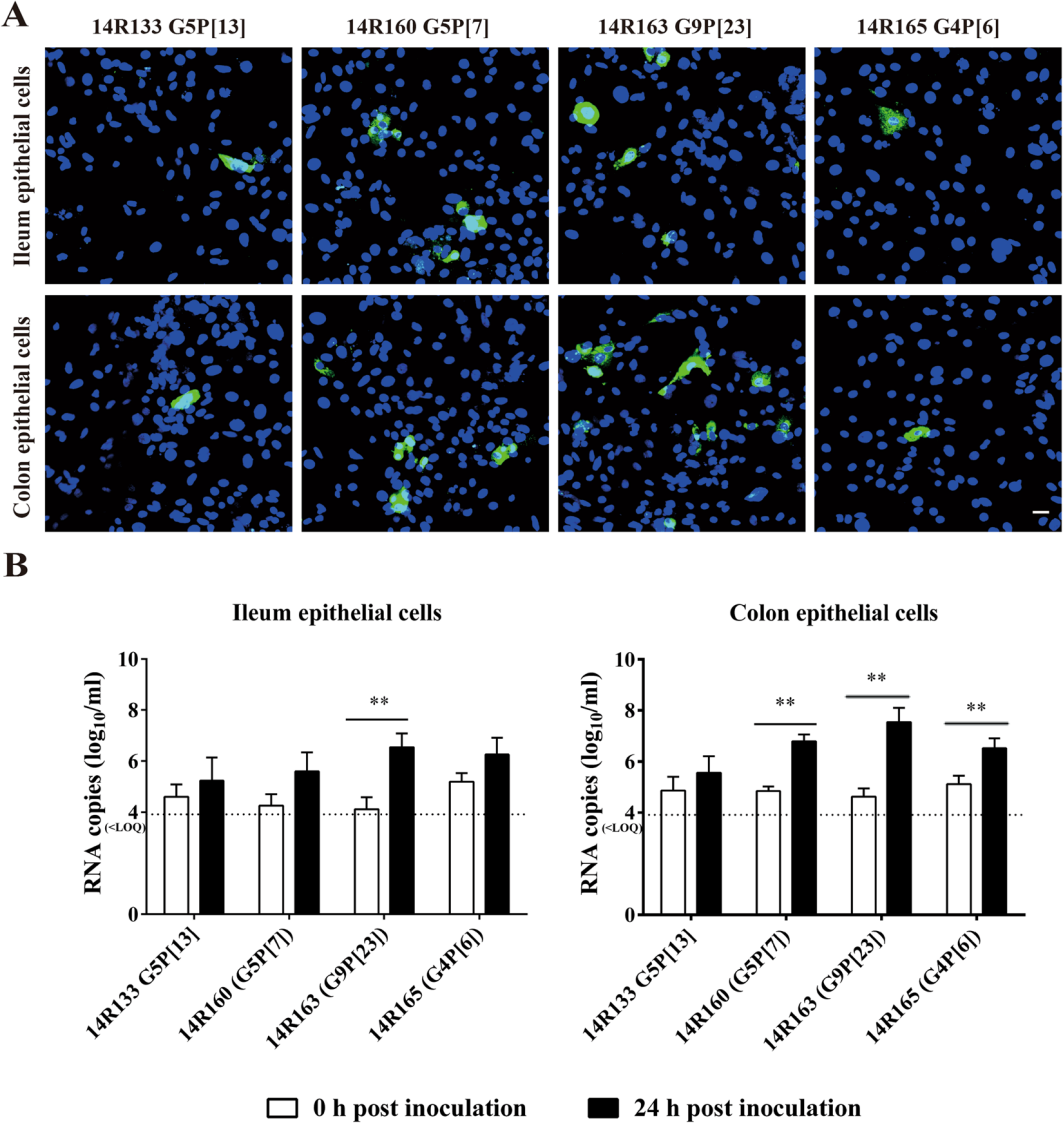
Susceptibility of porcine primary enterocytes to different rotavirus genotypes present in fecal suspension of diarrheic pigs. The infected cells were visualized by immunofluorescence staining at 24 h post inoculation (**A**). Viral RNA titer with supernatant of co-cultured primary porcine enterocytes inoculated with different rotavirus fecal suspensions at 0 h and 24 h post inoculation (**B**). Scale bar: 25 µm. Data are expressed as mean ± standard deviation of the results of 3 separate experiments. Statistically significant differences in comparison with the data from 0 h p.i. are represented as *P <0.05 or **P <0.01. <LOQ: below limit of quantification.

A major restriction of rotavirus research is the lack of cell cultures supporting the growth of different genotypes of field strains. Therefore, the susceptibility of primary porcine enterocytes co-cultured with myofibroblasts to four different genotypes of rotavirus A contained in fecal suspensions was tested by RT-qPCR (Fig. 7). The results showed that these four genotypes of rotavirus could infect both ileum and colon epithelial cells. Rotavirus 14R163 (G9P[23]) strain demonstrated the highest infectivity to primary enterocytes, followed by the 14R160 (G5P[7]) strain. For rotavirus 14R165 (G4P[6]) strain, an increase of more than 10 fold RNA copies/ml was detected at 24 h post inoculation of ileum and colon epithelial cells. Ileum and colon epithelial cells were less susceptible to rotavirus 14R133 (G5P[13]) strain. These results demonstrated that the primary enterocytes could be infected by rotavirus field strains containing different genotypes but that the susceptibility of enterocytes to rotavirus differed among the different genotypes.

### Susceptibility of co-cultured enterocytes to transmissible gastroenteritis virus

**Figure 8:**
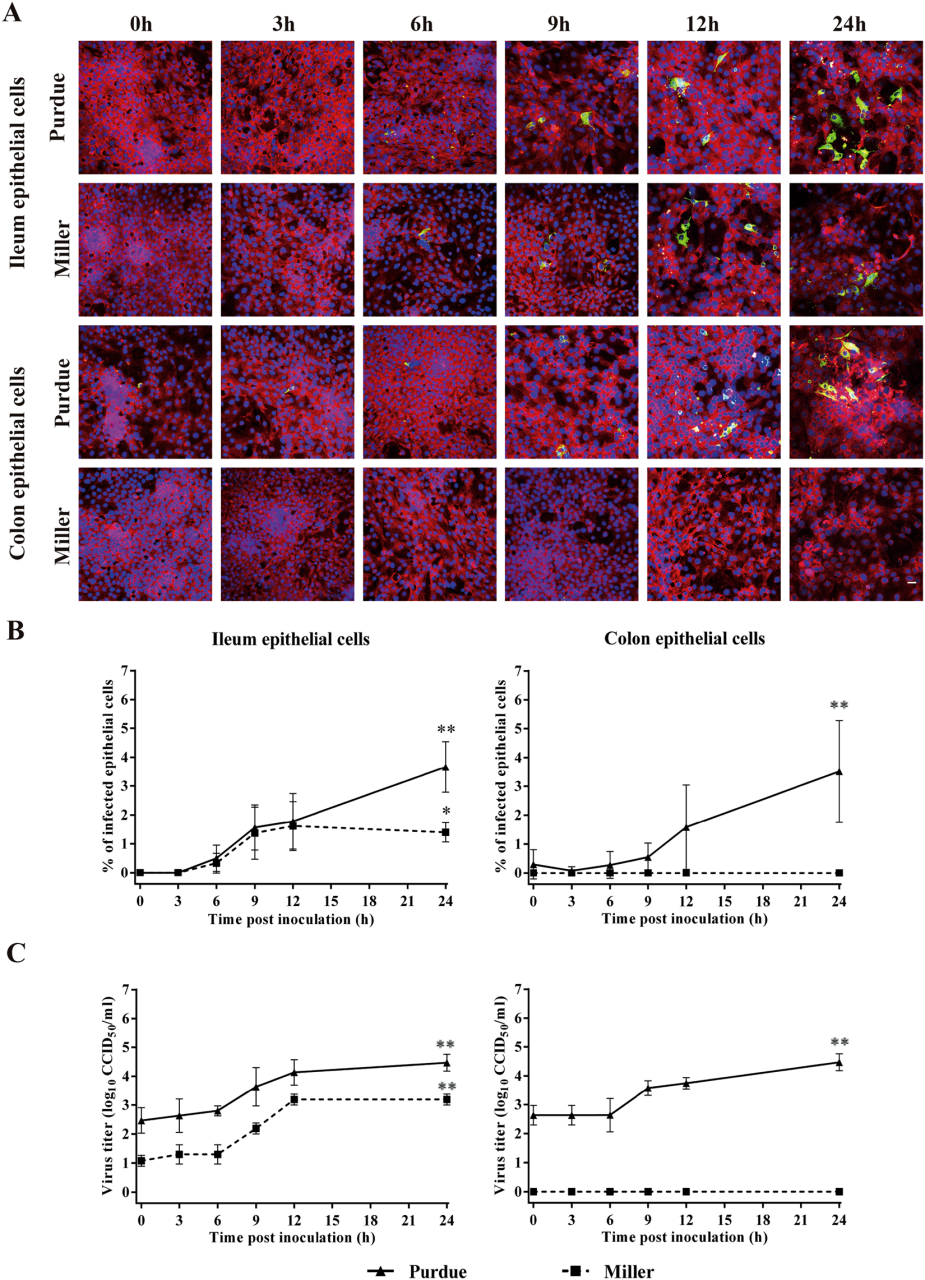
Kinetics of TGEV replication in ileum and colon epithelial cells co-cultured with myofibroblasts. Three days post co-cultivation, cells were inoculated with TGEV strains Purdue and Miller at an m.o.i. = 1. At different time points post inoculation, (**A**) infected cells were visualized by immunofluorescence staining (red represents cytokeratin and green represents TGEV antigen positive cells) and (**B**) the percentage of infected epithelial cells and (**C**) virus titers in the supernatant were determined. Scale bar: 25 µm. Data are expressed as mean ± standard deviation of the results of 3 separate experiments. Statistically significant differences in comparison with the data from 0 h p.i. are represented as *P <0.05 or **P <0.01.

Next, the susceptibility of primary enterocytes in co-cultures with myofibroblasts was tested for the TGEV Purdue and Miller strains. TGEV antigen expression kinetics were assessed in primary enterocytes (Fig. 8). TGEV Purdue infected both ileum and colon epithelial cells. At 24 h p.i, the Purdue strain had infected 3.7 ± 0.9% and 3.5 ± 1.7% of the ileum epithelial cells and colon epithelial cells, respectively, and the virus titer in both ileum and colon epithelial cells increased to 4.5 ± 0.3 CCID_50_/ml. TGEV Miller only infected ileum epithelial cells. The highest infection (1.6 ± 0.8%) appeared at 12 h p.i. No TGEV antigens of Miller strain were found in colon epithelial cells on 24 h post inoculation.

### Susceptibility of co-cultured ileum epithelial cells to porcine epidemic diarrhea virus

**Figure 9:**
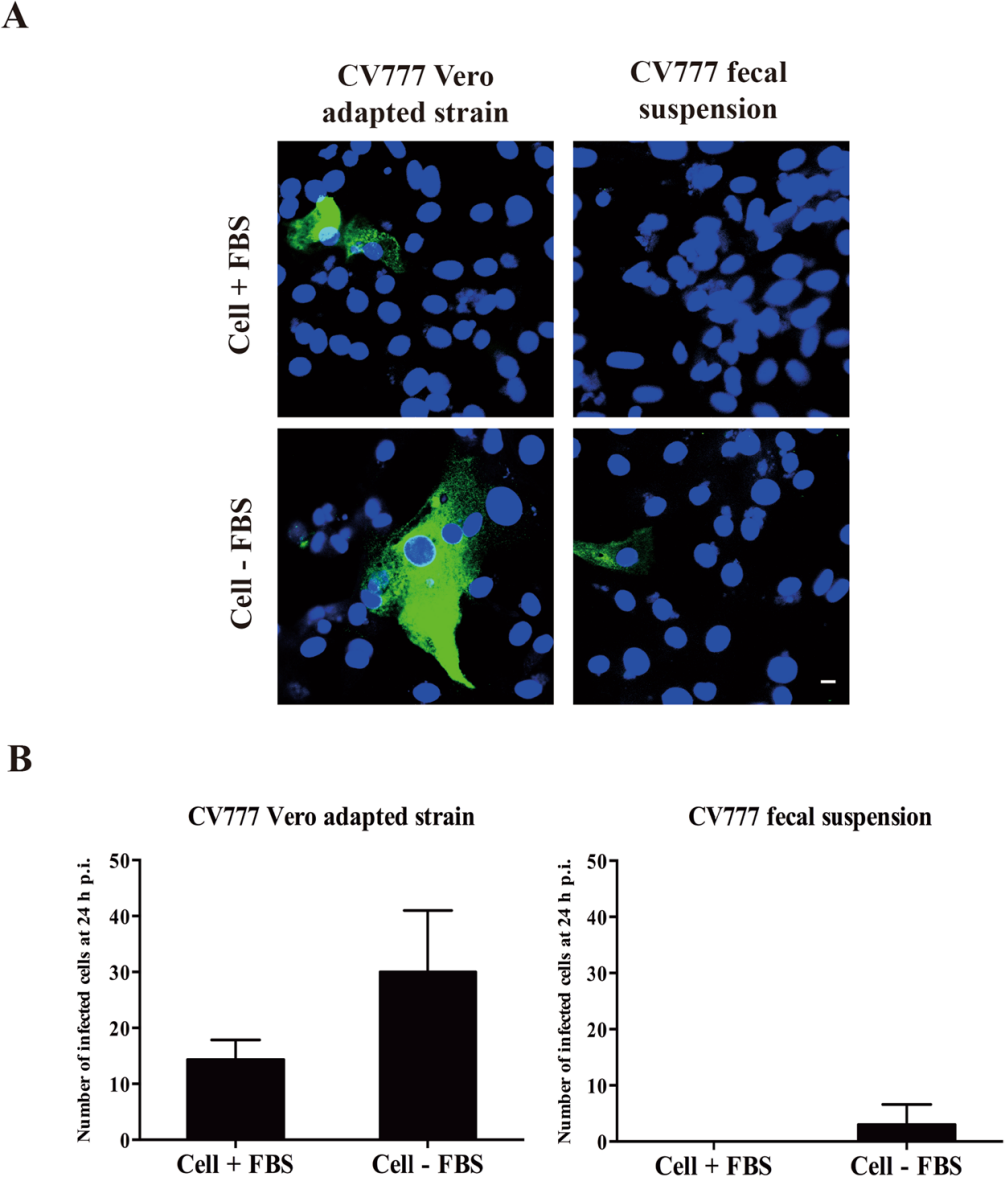
Infection of ileum epithelial cells co-cultured with myofibroblasts with PEDVCV777. Twenty-four hours post isolation, cells were refreshed with culture medium with/without FBS. Three days post co-cultivation, cells were inoculated with PEDV CV777 Vero adapted strain containing 10^4.9^ CCID50 or with fecal material containing 10^7^ viral RNA copies/ml of wild type CV777. Twenty-four hours post inoculation, the infection was visualized by immunofluorescence staining (**A**) and the total number of infected epithelial cells in a 24 well plate was determined (**B**). Scale bar: 10 µm. Data are expressed as mean ± standard deviation of the results of 3 separate experiments.

The PEDV CV777 Vero adapted strain and PEDV CV777 positive fecal suspensions were used to infect co-cultured primary ileum epithelial cells to confirm the usability of primary enterocytes. Twenty-four hours post inoculation, viral antigens of Vero adapted CV777 were observed in a low number of ileum epithelial cells cultured with/without FBS. Two times more infection was found in epithelial cells cultured without FBS. For CV777 fecal suspension, virus infection was only observed in ileum epithelial cells which were cultured without FBS (Fig. 9).

## Discussion

In this study, a co-culture system of porcine ileum subepithelial myofibroblasts with porcine small (ileum) and large (colon) intestinal epithelial cells was established and the use of this co-culture system for enteric virus research was assessed. The cross talk between epithelium and subepithelial myofibroblasts has been reported to promote epithelial cell proliferation and differentiation in both mice and humans. In this study, a myofibroblast cell line was established from porcine ileum which was identified by the presence of α-smooth muscle actin, vimentin and fibronectin. The *in vivo* distribution of epithelial cells and myofibroblasts shows that a lot of myofibroblasts directly grow underneath the epithelium in porcine ileum and that myofibroblasts form an integral line along colon crypts. This initial contact may be an important factor for the support of myofibroblasts towards epithelial cells.

At present, many mechanical and enzymatic seperation methods have been used for the isolation of intestinal epithelial cells from human, mice, rat, bovine, porcine and feline intestines. However, the successful cultivation of intestinal epithelial cells still poses a big challenge because of the rapid death/apoptosis of isolated epithelial cells which *in vivo* renew every 2–3 days. This apoptosis may be triggered by the disruption of the epithelial cell contact with extracellular matrix. A dispase and collagenase combination was used for epithelial cell isolation in the present study, which preserves more cell-to-cell interactions and reduce the damage of cell-matrix adhesions^29^. The contamination with stromal cells is a huge problem for epithelial cell cultivation. In order to decrease the contamination with mesenchymal cells, we removed these cells by D-sorbitol density centrifugation and plastic adhesion for 2 hours. According to the specific property that stromal cells attach to plates faster than epithelial cells, most stromal cells were separated from epithelial cells after 2 hours incubation. In the presence of ileum myofibroblasts, both ileum and colon epithelial cells are growing longer than one week and maintain their polygonal, cobblestone-like morphology. In the absence of myofibroblasts, epithelial cells died after 2–3 days, even when supplemented with 20% conditioned medium collected from myofibroblast cultures. Our data indicate that the supporting effect of myofibroblasts for epithelial cell growth is very dependent on the direct contact between these two cell types. We also demonstrated that myofibroblasts not only support the growth of intestinal epithelial cells from newborn piglets, but also the epithelial cells of 6 weeks old pigs (data not shown), which confirms the important role of myofibroblasts on epithelial cell proliferation independently of the age of the donor. The epithelial cells in co-cultures were identified by the presence of cytokeratin which is regarded as an important marker of epithelial cells. Most of the cells (>90%) preserved their epithelial nature with a positive staining of cytokeratin after 3 days of co-cultivation. Remarkably, the myofibroblasts clustered into aggregates in this co-culture system. It seems that myofibroblasts retracted into aggregates during the expansion of epithelial cells growth. In earlier reports, it was shown that myofibroblasts can migrate to wound tissue and demonstrate high contractile activities to generate tissue contractures, which help wound healing and organ remodeling by secretion of extracellular matrix proteins and exerting strong contraction force^30–32^. In addition, human and porcine myofibroblasts express S100A4 proteins which have been demonstrated to be implicated in cancer cell migration^30,33^. Taken together all this information, we hypothesize that myofibroblasts first secrete extracellular matrix proteins, such as collagen and laminin, coordinating the attachment and proliferation of epithelial cells and migration of myofibroblasts in clusters. Epithelial cells co-cultured with myofibroblasts showed microvilli after 3 days of co-cultivation which is in accordance with the reported data that myofibroblasts not only support the growth of epithelial cells, but also stimulate the differentiation of epithelial cells^34^.

Rotavirus research is hampered by the lack of susceptible enterocyte cell lines. Although some cell lines, including MA104, Marc, IPEC-J2 and Caco-2 cells^35–37^ are susceptible to some rotavirus strains, a lot of genotypes of rotavirus, such as P[4], P[6], P[8], P[13] do not grow efficiently in these cell lines. Therefore, enterocyte cultures are an essential tool to investigate rotavirus-cell interaction. In this study, the susceptibility of primary enterocytes to different rotavirus genotypes were explored. Rotavirus RVA/Pig-tc/BEL/RV277/1977/G1P[7] strain, which was first isolated in Belgium in 1977 from a pool of watery diarrhea of 3 pigs^38^, is a typical G1 porcine rotavirus. G1 is the most common VP7 genotype of human group A rotavirus, but is rarely found in porcine rotavirus strains^39^. It is suggested that all human G1 VP7 genes originate from porcine rotavirus transmission to humans and that this interspecies transmission was followed by human-to-human transmissions^40^. Although rotavirus was reported to have an exclusive tropism for small intestinal enterocytes^41^, rotavirus RVA/Pig-tc/BEL/RV277/1977/G1P[7] strain could infect both primary ileum and colon epithelial cells with a trypsin treatment. The antigen expression kinetics did not show significant differences in cell tropism of this rotavirus strain. Studies showed that some animal group A rotaviruses, especially porcine rotaviruses recognize sialic acid as host receptor for virus attachment. On porcine small and large intestinal epithelium, sialic acid receptors were clearly detected and colon crypts showed even a greater abundance of sialic acid than small intestines^42^. Rotavirus genotype P[7] has been classified as a sialic acid-dependent genotype^43^, which may explain why rotavirus G1P[7] strain could also infect colon epithelial cells *in vitro*. Both ileum and colon epithelial cells were also susceptible to four fecal suspensions containing different rotavirus genotypes (G5P[13], G5P[7], G9P[23] and G4P[6]). G5P[7] and G9P[23] rotaviruses, which are the predominant G/P genotype combination of circulating rotaviruses in Belgium, were more infectious than the other two strains, which was also observed on MA104 cells^8^. The higher infectivity in enterocytes *in vitro* may explain the wide prevalence of these genotypes in the field. Rotavirus P[6] genotype is one of the major human rotavirus genotypes. It is associated with symptomatic infection in children and is frequently detected in Africa. In pigs, P[6] rotaviruses are also regularly detected and most of these strains display a high genetic similarity to human P[6] rotavirus strains^8,44^. Animal rotaviruses are regarded as a potential reservoir for the genetic and antigenic diversity of human rotaviruses and interspecies transmission from swine to humans has been reported increasingly for P[6] genotype. Eight Hungarian human G4P[6] rotavirus strains were supposed to originate from pigs by independent events of zoonotic transmission^10^. Therefore, studying porcine rotavirus is a key step to deeply understand the evolution of human rotavirus. Recent studies demonstrated that P[6] rotavirus strains recognize human histo-blood group antigens (HBGAs), which explains why most human rotavirus strains cannot be cultured in MA104 cells. In our study, rotavirus G4P[6] could infect both ileum and colon epithelial cells which demonstrates that the primary enterocytes contain the cellular receptor for rotavirus P[6], likely containing the right HBGAs. This receptor is still not identified. Rotavirus almost exclusively infects mature enterocytes of the small intestinal villi *in vivo*^41^, while *in vitro*, the large intestinal epithelial cells could also be infected, which leads us to think that other factors in the large intestines block the rotavirus infection of colon epithelial cells *in vivo*. In the enterohepatic circulation, bile salts are synthesized in the liver, travel to the gall bladder and are secreted into the descending part of the duodenum helping in the digestion of fats and other substances. Up to 95% of secreted bile salts are collected in the ileum and return back to the liver via the portal system. We hypothesize that the lack of bile salts in the lumen of the large intestines may be the reason why rotavirus cannot infect the epithelial cells of large intestines *in vivo*. In order to test this hypothesis, we collected bile from the porcine gall bladder and used it for the infection of rotavirus to primary ileum epithelial cells. The results show that the infectivity of rotavirus in ileum epithelial cells increased after treatment with bile (Supplementary Fig. S1), which is in accordance with the results that bile/bile acids are essential for porcine enteric calicivirus, hepatitis C virus and human norovirus infection in cells^22,45,46^.

Porcine epidemic diarrhea virus and transmissible gastroenteritis virus primarily replicate in the villous enterocytes of the small intestine. Porcine aminopeptidase N (pAPN), one of the type II cell surface metalloproteases, was reported to be a cellular receptor for both PEDV and TGEV^47–49^. Although these two viruses bear similarities in structure and disease, they are clearly distinct. *In vitro*, PEDV does not grow in continuous cell cultures which are permissive to TGEV. Therefore, it is of interest to investigate the susceptibility of the target cell, primary porcine enterocytes, to both PEDV and TGEV. Interestingly, we found that TGEV Purdue can infect both ileum and colon epithelial cells, whereas the virulent Miller strain only infects ileum epithelial cells and that the infectivity of the Purdue strain is much higher than the Miller strain in ileum epithelial cells. The Purdue and Miller strains are two virulent American TGEV strains, which originally caused 100% mortality of less than 2-weeks-old piglets due to the lytic infection of enterocytes. The Purdue strain used in this study has been passaged 114 times in primary porcine kidney cells and then adapted to grow in ST cells by one more passage^50^. A nucleotide mutation (T to G at nucleotide position 1753) of the Purdue S protein, which causes a serine (S) to alanine (A) mutation at aa 585 was reported^18^. This mutation is present in the domain of the S protein encoded by nucleotide 1518–2184 which is used for the cell receptor pAPN recognition^51^. This mutation may make the virus more suitable to grow in pAPN negative cultures/primary porcine kidney cells due to the recognition of other cell receptors. It may also be responsible for the growth of the Purdue strain in primary porcine colon epithelial cells and porcine myofibroblasts which are both negative for pAPN (Supplementary Fig. S2). Due to the absence of pAPN, the Miller strain cannot infect the primary colon epithelial cells. PEDV CV777 strain has been adapted to grow in the APN negative Vero cells by several blind passages and has been widely used for PEDV research. We found that primary ileum epithelial cells were more susceptible to the CV777 Vero adapted strain when the cells were grown in absence of FBS and the CV777 fecal suspension only infected the cells which were cultured without FBS. FBS is usually added in culture medium for cell growth because it contains various biological factors. But its compositional complexity also causes a lot of side-effects, such as the inhibition of cell differentiation and virus replication. FBS may inhibit viral replication by blocking cellular proteins, as shown for another nidovirus porcine reproductive and respiratory syndrome virus (PRRSV)^52^. CV777 strain shows a lower infectivity in ileum epithelial cells cultured with FBS suggesting that FBS contains factors that interfere with PEDV infection or that without FBS the epithelial cells could differentiate better which makes them more susceptible to PEDV infection. The susceptibility of primary ileum epithelial cells to a PEDV fecal suspension represents a big step for further investigating PEDV field isolates.

In this study, the primary epithelial cells were susceptible to rota- and coronaviruses, however the infection efficiency was relatively low. This might be caused by the antiviral response of interferons, especially type III IFNs (IFN-λ). Interferons belong to the class of cytokines, which are made and released by host cells to trigger protective defenses to several pathogens, such as viruses, bacteria and parasites. Intestinal epithelial cells abundantly produce type III IFNs and elicit antiviral defenses in response to viral infections. Zhang and colleagues demonstrated that IFN-λ possesses a strong antiviral effect on PEDV replication in the porcine intestinal epithelial cell line IPEC-DQ, a subclone obtained from the nontransformed IPEC-J2 cells by limited serial dilutions^53^. A similar strong IFN-λ might have been rapidly induced in the currently described co-cultures. Furthermore, rotavirus, PEDV and TGEV infect mature villous epithelial cells. In our co-culture system, not only the mature villous epithelial cells were cultured but likely also the less differentiated intestinal crypt epithelial cells. These undifferentiated crypt epithelial cells might cause the low infection efficiency. In future work, efforts will be made to stimulate the differentiation of primary epithelial cells and control the IFN antiviral response in order to increase the infection efficiency.

In conclusion, porcine enterocyte/myofibroblast co-cultures were successfully established in the present study, demonstrating that myofibroblasts are necessary for small and large intestinal epithelial cell attachment, proliferation and differentiation *in vitro*. Primary enterocytes were susceptible to both cell line adapted and non-adapted rota-and coronaviruses. This co-cultivation system will be a great assett in future research on rota-, coronavirus and other enteric viruses.

## Materials and Methods

### Ethical statement

Euthanizing piglets was done in agreement with the European legislation on animal experiments. All experimental procedures were approved by the Local Ethical Committee of the Faculty of Veterinary Medicine, Ghent University, and all methods were carried out in accordance with the approved guidelines.

### Piglets and virus samples

Healthy conventional 3-day-old suckling piglets were purchased from a conventional pig farm. Using tissues of euthanized animals was in accordance of the requirements of the Local Ethical Committee of the Faculty of Veterinary Medicine. The piglets were euthanized by intravenous injection of 20% sodium Pentobarbital (1 ml/1.5 kg, Kela Laboratories, Hoogstraten, Belgium). Rotavirus RVA/Pig-tc/BEL/RV277/1977/G1P[7] strain grown on MA104 cells, TGEV Purdue and Miller strain grown on ST cells and PEDV CV777 strain grown on Vero cells were used in this study. In addition, four diarrheic fecal suspensions containing rotavirus of suckling pigs less than 2 weeks old (Table 1) and PEDV CV777 fecal suspension from an experimentally inoculated 3-day-old sucking piglet were also included. Twenty percent fecal suspensions were prepared in phosphate buffered saline (PBS) containing 1000 U/ml penicillin (Continental Pharma, Puurs, Belgium), 1 mg/ml streptomycin (Certa, Braine l’Alleud, Belgium), 1 mg/ml gentamicin (Gibco BRL, Merelbeke, Belgium) and 0.01% v/v fungizone (Bristol-Myers Squibb, Braine l’Alleud, Belgium)^7^.

**Table 1:** RT-qPCR diagnosis of rotavirus fecal suspensions^7^.

Sample	G/P genotype	RVA qPCR load*	RVC qPCR load
RVA/Pig-wt/BEL/14R133/2014/G5P[13]	G5P[13]	9.21	Negative
RVA/Pig-wt/BEL/14R160/2014/G5P[7]	G5P[7]	10.63	Negative
RVA/Pig-wt/BEL/14R163/2014/G9P[23]	G9P[23]	10.63	Negative
RVA/Pig-wt/BEL/14R165/2014/G4P[6]	G4P[6]	9.27	Negative

*Is RT-qPCR titer: log10 copies/g faeces.

### Establishment of porcine intestinal subepithelial myofibroblasts

The ileum was collected from a euthanized 3-day-old piglet and brought in ice-cold Dulbecco’s Modified Eagle Medium (DMEM; Gibco BRL, Merelbeke, Belgium), containing 100 U/ml penicillin, 0.1 mg/ml streptomycin, 0.1 mg/ml gentamycin (flushing medium) and 10% fetal bovine serum (FBS; Gibco BRL). Subsequently, the intestine was cut into 6–8 cm long segments and turned inside-out, mucosal side facing outwards. Intestinal contents were removed by one washing with ice-cold flushing medium containing 10% FBS and two washings with flushing medium without FBS. The intestinal segments were incubated in PBS containing 1 mM EDTA and shaken at 150 rpm/min for 30 min at 37 °C. The EDTA suspension was removed and this EDTA incubation step was repeated 3 times. Next, intestinal segments were incubated in DMEM containing dispase II (1.2 mg/ml, Sigma, St. Louis, MO, USA) and collagenase I (0.4 mg/ml, Invitrogen, Paisley, UK) for 30 min at 37 °C. Subsequently, the digested mucosa was gently scraped with a sterile scalpel blade, scrapings were collected and incubated in DMEM containing dispase II (1.2 mg/ml) for 10 min whilst pipetting. After centrifugation for 4 min at 140 × *g* and 4 °C, the pellet was resuspended in DMEM containing 2% D-sorbitol (Sigma) and 2.5% FBS, and centrifuged at 50 × *g* for 4 min. The pellet was resuspended in DMEM/F-12 (Gibco BRL) culture medium supplemented with 100 U/ml penicillin, 0.1 mg/ml streptomycin, 2% FBS, and 1% non-essential amino acids (Gibco BRL) and incubated at 37 °C and 5% CO_2_. Culture medium was refreshed on day 2, afterwards medium was changed every 3 days. Morphology of the cells was evaluated daily by light microscopy (Olympus). Once myofibroblasts (cobblestone-like clusters) grew into big clusters (≈200 cells), they were marked and other cells (e.g. epithelial cells, fibroblasts) were removed by scraping. Then the cobblestone-like clusters were detached by trypsinization with 5 μg/ml trypsin-0.01% EDTA, and sub-cultured in a 24-well plate (split ratio 1:2) with culture medium containing 10% FBS and evaluated daily for cobblestone-like features by light microscopy. Subsequently, the cobblestone-like cells were digested by trypsinization and further expanded in flasks to generate a long-term semi-continuous culture.

### Characterization of isolated myofibroblasts

To characterize the obtained primary cells, immunofluorescence stainings were performed to visualize cytokeratin, vimentin, α-smooth muscle actin, fibronectin, desmin and sucrase-isomaltase. A third passage of cells was fixed with 4% paraformaldehyde for 10 min at room temperature (RT) followed by permeabilization with 0.1% Triton X-100 for 5 min at RT. The cells were respectively incubated with mouse monoclonal anti-human cytokeratin antibodies (Dako, Denmark A/S), mouse monoclonal anti-human vimentin antibodies (Bio-Rad), mouse monoclonal anti-human α-smooth muscle actin antibodies (Dako), mouse monoclonal anti-human desmin antibodies (Dako) sheep polyclonal anti-human fibronectin antibodies (Bio-Rad) or mouse monoclonal anti-human sucrase-isomaltase (Santa Cruz) for 1 h at 37 °C. Afterwards, cells were washed and incubated with goat anti-mouse-IgG FITC labeled antibodies (Molecular Probes) or rabbit anti-sheep-IgG FITC labeled antibodies (Molecular Probes) for 1 h at 37 °C. Nuclei were stained with Hoechst 33342 (Molecular Probes) for 10 min at RT. The slides were mounted using glycerin solution with 2.5% 1,4-diazabicyclo[2.2.2]octane (Janssen Chimica, Beerse, Belgium) and analyzed using fluorescence microscopy (DM B fluorescence microscope, Leica Microsystems GmbH, Heidelberg, Germany).

### Isolation of primary porcine enterocytes using myofibroblasts as supporting cells

#### Preparation of supporting cells

Two days before enterocyte isolation, myofibroblasts were seeded in 24-well plates at a density of 60000 cells/ml in DMEM/F-12 culture medium containing 100 U/ml penicillin, 0.1 mg/ml streptomycin, and 10% FBS, and 1% non-essential amino acids and cultured at 37 °C and 5% CO_2_. Monolayers of myofibroblasts were used as support layer for enterocytes growth and differentiation.

#### Preparation of conditioned medium collected from myofibroblasts

When myofibroblasts grew to 80% confluency, their medium was refreshed by DMEM/F-12 culture medium. Next, the refreshed medium was collected after 24 h, centrifuged at 2000 × *g* for 10 min and the supernatant was collected and used as conditioned medium. The conditioned medium was stored at −70 °C until later use.

#### Isolation of porcine enterocytes

After euthanasia, around 10 cm long segments of ileum and colon were removed from a piglet. The intestinal segments were turned inside-out, mucosal side facing outwards. The intestinal contents were removed by one washing with ice-cold flushing medium with 10% FBS and two washings with ice-cold flushing medium without FBS. In order to fully expand the contact area of intestinal mucosa layer to digestion enzymes, one side of the intestinal piece was closed with a surgical clamp and the lumen was filled with warm Ca^2+^- and Mg^2+^-enriched PBS containing penicillin and streptomycin. Then, the other side was also closed with a surgical clamp. The intestinal mucosa was digested in DMEM containing dispase II (1.2 mg/ml) and collagenase I (0.4 mg/ml) for 20 min at 37 °C. Then, the PBS was released from the lumen by removing one clamp. The mucosa was gently scraped with a sterile scalpel blade. Afterwards, the intestinal lumen was filled again with PBS and the mucosa was digested in DMEM containing dispase II (1.2 mg/ml) and collagenase I (0.4 mg/ml) again for another 20 min (ileum) or 40 min (colon) at 37 °C. Subsequently, the digested mucosa was deeply scraped with a sterile scalpel blade. The scrapings were incubated in DMEM containing dispase II (1.2 mg/ml) for 10 min whilst pipetting. After centrifugation (140 × *g*, 4 min at 4 °C) the pellet was resuspended in DMEM containing 2% D-sorbitol and 2.5% FBS and centrifuged at 50 × *g* for 4 min at 4 °C to separate single stromal cells. This sorbitol process was repeated at least 5 times. Afterwards, the pellet was resuspended in DMEM supplemented with antibiotics and filtered using a 100 μm cell strainer (Falcon). After centrifugation (338 × *g*, 10 min), the pellet was finally resuspended in DMEM/F12 supplemented with 100 U/ml penicillin, 0.1 mg/ml streptomycin, 0.1 mg/ml gentamycin, 10% FBS, 0.01% fungizone, 10 ng/ml epidermal growth factor (Sigma), 1% insulin-transferrin-selenium-ethanolamine (Gibco BRL) and 1% non-essential amino acids and seeded in 6-well plates in order to let the mesenchymal cells attach and separate enterocytes from mesenchymal cells. After 2 h incubation at 37 °C and 5% CO_2_, the non-adherent cell clusters in the 6-well plates were collected and reseeded on top of the monolayer of myofibroblasts. To confirm the support effect of myofibroblasts, cell clusters were also seeded on porcine collagen type I/III (Gentaur, Kampenhout) coated plates and cultured with/without 20% conditioned medium. The cells were further incubated at 37 °C and 5% CO_2_ and the medium was refreshed every 2 days. Morphological features of primary enterocytes were evaluated by light microscopy (Olympus).

### Characterization of primary enterocytes co-cultured with myofibroblasts

To characterize the origin of enterocytes which were co-cultured with myofibroblasts, immunofluorescence staining was performed against cytokeratin and vimentin. Three days post isolation, co-cultured enterocytes grown on coverslips were fixed with 4% paraformaldehyde for 10 min at RT followed by permeabilization with 0.1% Triton X-100 for 5 min at RT. The cells were incubated with mouse monoclonal anti-human cytokeratin or mouse monoclonal anti-human vimentin antibodies containing 10% goat serum for 1 h at 37 °C, followed by goat anti-mouse-IgG FITC labeled antibodies for 1 h at 37 °C. Nuclei were stained with Hoechst for 10 min at RT. The percentage of cytokeratin positive cells were analyzed by fluorescence microscopy (Leica Microsystems GmbH).

### Distribution of epithelial cells and myofibroblasts in the intestine of a 3-day-old piglet

Immediately after euthanasia of a 3-day-old piglet, 8 mm square pieces of the ileum and colon were collected. Tissues were embedded in Methocel (Fluka, Sigma) and 10 μm thick cryosections were made. Immunofluorescence staining with markers for epithelial cells and myofibroblasts were performed. In brief, cryosections of ileum and colon tissues were fixed with methanol for 20 min at −20 °C and then incubated with mouse monoclonal anti-human α-smooth muscle actin antibodies for 1 h at 37 °C, followed by goat anti-mouse-IgG FITC labeled antibodies for 1 h at 37 °C. Afterwards, the sections were incubated with rabbit polyclonal anti-bovine cytokeratin antibodies, followed by goat anti-rabbit-IgG Texas Red labelled antibodies for 1 h at 37 °C. After washing, nuclei were stained with Hoechst for 10 min at RT. The slides were analyzed by fluorescence microscopy.

### Scanning electron microscopy

In order to determine the differentiation status of intestinal epithelial cells, the presence of microvilli was assessed by scanning electron microscopy (SEM). The protocol for SEM was performed as described by Glorieux and colleagues^54^. Tissue samples (ileum and colon from 3-day-old piglet) and cell samples (porcine enterocytes 3 days post co-cultivation) were fixed in HEPES-buffered glutaric aldehyde for 24 hours. Then, the samples were treated with 1% osmiumtetroxide for 2 hours at RT, followed by ascending grades of alcohol dehydration. In order to avoid the water vaporization obstructing the electron beam and interfering with image clarity, the dehydrated samples were transferred to a critical point drier (CPD, Bal-tec, Balzers, Liechtenstein) for complete drying. Finally, the dried samples were mounted on a metal stub and sputter-coated with platinum. The microvilli of all the samples were acquired with a JEOL JSM 5600 LV scanning electron microscope (JEOL Ltd., Tokyo, Japan).

### Susceptibility of co-cultured enterocytes to porcine rotavirus

To evaluate the susceptibility of porcine primary enterocytes co-cultured with myofibroblasts to porcine rotavirus, a third passage of rotavirus RVA/Pig-tc/BEL/RV277/1977/G1P[7] strain was used. Before inoculation, virus was pretreated with 5 μg/ml trypsin for 30 min at 37 °C to enhance virus infectivity. After three washes with DMEM, 3 days co-cultured cells were inoculated with 200 μl pretreated virus at a multiplicity of infection (m.o.i.) of 1. After 1 h inoculation at 37 °C and 5% CO_2_, the inoculum was removed and cells were washed 3 times with warmed DMEM. The cells were further incubated with DMEM/F12 supplemented with 100 U/ml penicillin, 0.1 mg/ml streptomycin, 0.1 mg/ml gentamycin, 10 ng/ml epidermal growth factor, 1% insulin-transferrin-selenium-ethanolamine, 1% non-essential amino acids and 1 μg/ml trypsin at 37 °C and 5% CO_2_. Twenty-four hours post inoculation, cells were fixed with 4% paraformaldehyde for 10 min and permeabilized with 0.1% Triton X-100 for 5 min at RT. Double-immunostainings against rotavirus antigens and cytokeratin were performed to specifically visualize the infected enterocytes. The cells were incubated with polyclonal guinea pig anti-monkey rotavirus SA-11 VP6 antibodies^55^ (kindly provided by John Patton), containing 10% negative goat serum for 1 h at 37 °C, followed by goat anti-guinea pig-IgG FITC labelled antibodies (Southern Biotech) for 1 h at 37 °C. Afterwards, cells were incubated 1 h at 37 °C with monoclonal anti-human cytokeratin antibodies, followed by 1 h at 37 °C with goat anti-mouse-IgG Texas Red labelled antibodies (Molecular Probes). Nuclei were stained with Hoechst for 10 min at RT. The infected enterocytes were visualized by fluorescence microscopy.

### Replication of MA104 grown rotavirus in co-cultured enterocytes

Three days post seeding, primary enterocytes co-cultured with myofibroblasts were inoculated with 200 μl rotavirus RVA/pig-tc/BEL/RV277/1977/G1P[7] at an m.o.i. of 1. After 1 h of inoculation (37 °C and 5% CO_2_), cells were washed 3 times with warm DMEM and further incubated in serum-free DMEM/F12 culture medium containing 1 μg/ml trypsin. At different time points (0, 3, 6, 9, 12 and 24 h) post inoculation, cells were fixed with 4% paraformaldehyde for 10 min and permeabilized with 0.1% Triton X-100 for 5 min at RT. Double-immunostainings were performed as described above and the percentage of infected enterocytes was counted by fluorescence microscopy. In addition, the supernatant and cells were collected at different time points (0, 3, 6, 9, 12 and 24 h) post inoculation for virus titration and qPCR quantification. Cell culture supernatant was collected and centrifuged for 5 min at 3756 × *g*. Supernatant was collected (extracellular virus) and the pellet was collected together with cells that were scraped with serum free DMEM/F12 culture medium (intracellular virus). The cells were lysed by 3 freeze-thaw cycles to release virus particles. The virus titer was determined using virus titration and RT-qPCR. Titration of intra- and extracellular virus was performed. Monolayers of MA104 cells growing in 96-well plates were inoculated with 10-fold dilution of supernatant and cells (10^−1^–10^−8^). Five days after inoculation, the cytopathogenic effect (CPE) was visualized using a light microscope and the cell culture infective dose (CCID_50_) was calculated using the formula of Reed and Muench^56^. For the determination of viral RNA copies, intra- and extracellular virus were treated with a cocktail of Benzonase (Novagen, Madison, USA) and homemade buffer (1 M Tris, 100 mM CaCl_2_ and 30 mM MgCl_2_, pH 8) at 37 °C for 2 h. After the EDTA treatment for Benzonase inactivation, total RNA was extracted using QIAamp Cador Pathogen Mini Kit (Qiagen) according to the manufacture’s instruction without addition of carrier RNA to the lysis buffer. RNA was denatured at 95 °C for 2 min and a 20 μl PCR mixture was used per reaction containing 10 μl precision OneStep^TM^ RT-qPCR Mastermix with SYBR Green and ROX, 0.8 μl (400 nM) forward primer (5′-TTTAAAAGCGCTACAGTGATG-3′), 0.1 μl (50 nM) reverse primer (5′-CGTTGCTTGAAGGTCGTGATT-3′) and 3 μl RNA or diluted standard RNA^8^. Reactions were performed of 10 min at 55 °C for a reverse transcription step and 2 min at 95 °C for an enzyme activation step which were followed by 40 cycles, 95 °C for 10 sec and 60 °C for 60 sec. Afterwards, a melt curve analysis was performed to check the specificity of the amplified products. All analyses were carried out in a StepOnePlus real-time PCR system (Applied Biosystems, Life Technologies Corporation, Carlsbad, CA, USA).

### Susceptibility of co-cultured enterocytes to different rotavirus genotypes present in fecal suspension of diarrheic pigs

Four fecal suspensions containing different genotypes of rotavirus collected from less than 2-week-old diarrheic suckling pigs were used to determine the susceptibility of porcine primary enterocytes to rotavirus field strains. These samples were collected at a private diagnostic laboratory for etiology diagnosis as described before^7^. Three days after co-cultivation, enterocytes were inoculated with these four fecal suspensions at 10^7^ viral RNA copies/ml using the method described above. The supernatant was harvested at 0 h and 24 h post inoculation. RT-qPCR was performed to determine the increased number of viral RNA copies.

### Susceptibility of co-cultured enterocytes to transmissible gastroenteritis virus

Three days post seeding, monolayers of primary enterocytes co-cultured with myofibroblasts were inoculated with 200 μl TGEV strains (Miller and Purdue) at an m.o.i. of 1. After 1 h inoculation at 37 °C and 5% CO_2_, cells were washed 3 times with warm DMEM. Serum free DMEM/F12 supplemented with 100 U/ml penicillin, 0.1 mg/ml streptomycin, 0.1 mg/ml gentamycin, 10 ng/ml epidermal growth factor, 1% insulin-transferrin-selenium-ethanolamine and 1% non-essential amino acids was added for further incubation. At different time points (0, 3, 6, 9, 12 and 24 h) post inoculation, cells were fixed with 4% paraformaldehyde for 10 min and the supernatant was collected. Double-immunostainings against both TGEV antigens and cytokeratin were performed to specifically visualize the infected enterocytes. The cells were permeabilized with 0.1% Triton X-100 for 5 min at RT and incubated with swine polyclonal anti TGEV antibodies containing 10% negative goat serum for 1 h at 37 °C, followed by goat anti-swine-IgG FITC labelled antibodies for 1 h at 37 °C. Afterwards, cells were incubated 1 h at 37 °C with mouse monoclonal anti-human cytokeratin antibodies, followed by 1 h at 37 °C with goat anti-mouse-IgG Texas Red labelled antibodies. Nuclei were stained with Hoechst for 10 min at RT. The percentage of infected enterocytes was determined by fluorescence microscopy. Virus titration of supernatant was performed using ST cells. Monolayers of ST cells were inoculated with 10-fold dilutions (10^0^–10^−7^) of supernatant. Five days after inoculation, cytopathogenic effect (CPE) was visualized and cell culture infective dose (CCID_50_) was calculated using the formula of Reed and Muench.

### Susceptibility of primary ileum epithelial cells to porcine epidemic diarrhea virus

Twenty-four hours post seeding, ileum epithelial cells were refreshed with culture medium with/without 10% FBS. Three days post cultivation, monolayers of primary ileum epithelial cells cultured with/without FCS were inoculated with 200 μl PEDV CV777 Vero adapted strain at 10^5.6^ CCID_50_/ml or 10^7^ viral RNA copies/ml of fecal suspension with 10 µg/ml trypsin. After 1 h inoculation at 37 °C and 5% CO_2_, cells were washed 3 times with warm DMEM and serum free DMEM/F12 supplemented with 100 U/ml penicillin, 0.1 mg/ml streptomycin, 0.1 mg/ml gentamycin, 10 ng/ml epidermal growth factor, 1% insulin-transferrin-selenium-ethanolamine and 1% non-essential amino acids was added for further incubation. Twenty-four hours post inoculation, cells were fixed with 4% paraformaldehyde for 10 min and permeabilized with 0.1% Triton X-100 for 5 min at RT. The cells were incubated with mouse monoclonal anti-pig PEDV antibodies containing 10% normal goat serum, followed by 1 h at 37 °C with goat anti-mouse-IgG FITC labelled antibodies. Nuclei were stained with Hoechst for 10 min at RT and the infection was analyzed by fluorescence microscopy.

## Electronic supplementary material

Supplementary information

## Data Availability

The datasets generated during and/or analysed during the current study are available from the corresponding author on reasonable request.
