# Supplementary material for: Establishment of porcine enterocyte/myofibroblast co-cultures for the growth of porcine rota- and coronaviruses
Source: Sci Rep. 2018 Oct 12;8:15195. doi: 10.1038/s41598-018-33305-1 (PMC6185943; doi:10.1038/s41598-018-33305-1)
Supplement: Supplementary file 1 — Supplementary information [file 41598_2018_33305_MOESM1_ESM.pdf]

Establishment of porcine enterocyte/myofibroblast co-cultures for the growth of porcine rota- and coronaviruses

Tingting Cui[1]*, Sebastiaan Theuns[1], Lowiese M.B. Desmarets[1], Jiexiong Xie[1], Gaëtan M.A. De Gryse[1], Bo Yang[1], Wim Van den Broeck[2], Hans J. Nauwynck[1]*

Supplementary documents

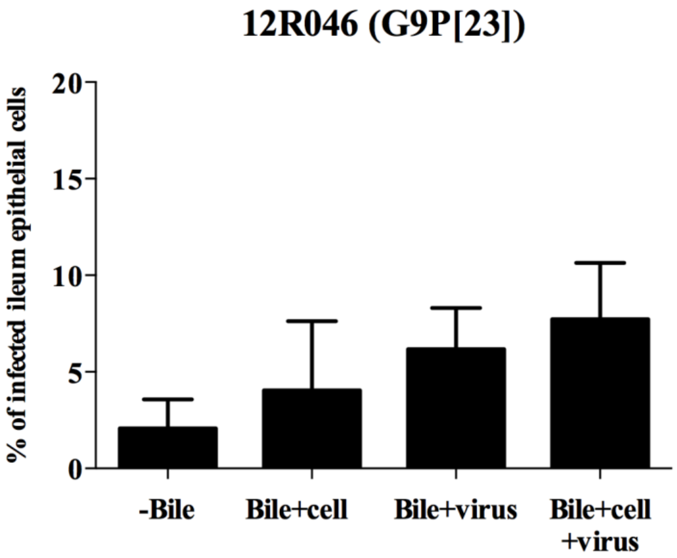

Figure S1. **Effect of bile treatment of rotavirus 12R046 strain on the replication in primary ileum epithelial cells.** Bile collected from the gallbladder of 12 weeks old pig was used to treat cells and virus separately and simultaneously. Percentage of infection was evaluated 24 h p.i. Data are expressed as means ± standard deviation of the results of 3 separate experiment.

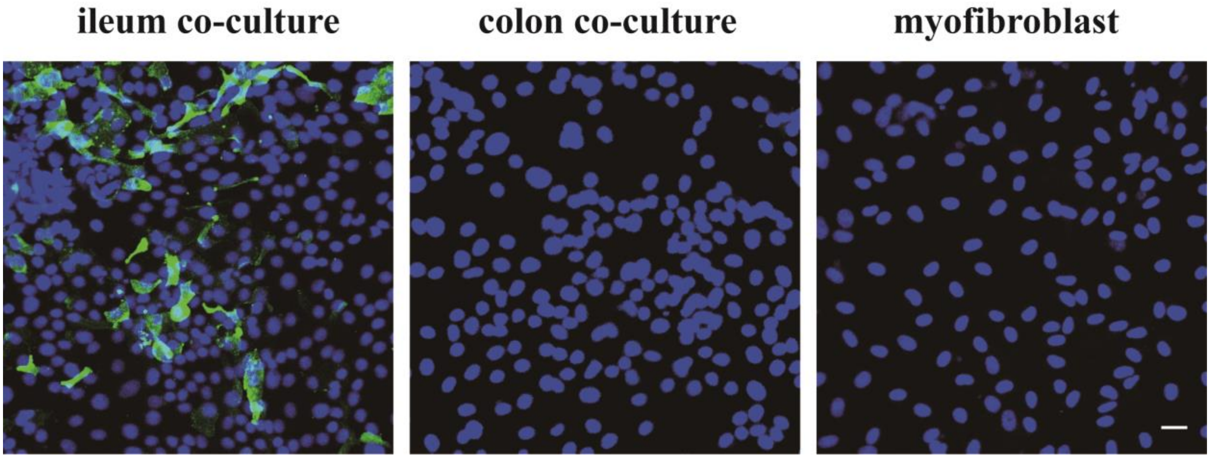

Figure S2. **Porcine aminopeptidase N (pAPN) expression of primary enterocytes and ileum myofibroblasts.** Three days post seeding, co-cultured enterocytes and myofibroblasts were stained with mouse monoclonal anti-porcine aminopeptidase N antibodies (green). Scale bar: 25 µm.
